# Production of a signal by irradiated cells which leads to a response in unirradiated cells characteristic of initiation of apoptosis

**DOI:** 10.1054/bjoc.2000.1433

**Published:** 2000-11

**Authors:** F M Lyng, C B Seymour, C Mothersill

**Affiliations:** Radiation Science Centre, Dublin Institute of Technology, Kevin Street, Dublin, 8, Ireland

**Keywords:** bystander effect, apoptosis, cell signalling, radiation effects

## Abstract

This study investigated the ability of medium from irradiated cells to induce early events in the apoptotic cascade, such as mobilization of intracellular calcium, loss of mitochondrial membrane potential and increase in reactive oxygen species, in cells which were never exposed to radiation. Medium from irradiated human keratinocytes was harvested and transferred to unirradiated keratinocytes. Endpoints characteristic of the initiation of apoptosis were monitored for a period of 24 h following medium transfer. Clonogenic survival was also measured. Rapid calcium fluxes (within 30 s), loss of mitochondrial membrane potential, increases in reactive oxygen species (from 6 h after medium transfer), an increase in the number of apoptotic cells (48 hours after medium transfer) and a marked reduction in clonogenic survival (after 9 days) were observed. There was no significant difference between medium generated by cells irradiated at 0.5 Gy or 5 Gy. The data suggest that initiating events in the apoptotic cascade were induced in unexposed cells by a signal produced by irradiated cells. © 2000 Cancer Research Campaign


*British Journal of Cancer* (2000) **83**(9), 1223–1230
© 2000 Cancer Research Campaign
doi: 10.1054/ bjoc.2000.1433, available online at http://www.idealibrary.com on IDEAL®

### Production of a signal by irradiated cells which leads to a response in unirradiated cells characteristic of initiation of apoptosis

**FM Lyng, CB Seymour and C Mothersill**

Radiation Science Centre, Dublin Institute of Technology, Kevin Street, Dublin 8, Ireland

**Summary** This study investigated the ability of medium from irradiated cells to induce early events in the apoptotic cascade, such as mobilization of intracellular calcium, loss of mitochondrial membrane potential and increase in reactive oxygen species, in cells which were never exposed to radiation. Medium from irradiated human keratinocytes was harvested and transferred to unirradiated keratinocytes. Endpoints characteristic of the initiation of apoptosis were monitored for a period of 24 h following medium transfer. Clonogenic survival was also measured. Rapid calcium fluxes (within 30 s), loss of mitochondrial membrane potential, increases in reactive oxygen species (from 6 h after medium transfer), an increase in the number of apoptotic cells (48 hours after medium transfer) and a marked reduction in clonogenic survival (after 9 days) were observed. There was no significant difference between medium generated by cells irradiated at 0.5 Gy or 5 Gy. The data suggest that initiating events in the apoptotic cascade were induced in unexposed cells by a signal produced by irradiated cells. © 2000 Cancer Research Campaign

**Keywords**: bystander effect; apoptosis; cell signalling; radiation effects

Considerable evidence has accumulated recently suggesting that a factor or signal can be released by irradiated cells which can affect unexposed cells in the field following high LET radiation (Nagasawa and Little, 1992; Deshpande et al, 1996; Azzam et al, 1998; Lorimore et al, 1998). Cells that are not even in the field can be affected by medium harvested from irradiated cells exposed to low doses of low LET radiation (Mothersill and Seymour, 1997, 1998; Seymour and Mothersill 1997, 2000). Low doses of α particles have been shown to lead to the formation of sister chromatid exchanges in 30–50% of the cell population despite the fact that only 1% of the cells' nuclei would have been traversed by an α particle (Nagasawa and Little, 1992; Deshpande et al, 1996). Extracellular factors produced by α particle-irradiation can cause sister chromatid exchanges (Emerit et al, 1967; Lloyd and Moquet, 1985; Lehnert and Goodwin, 1997) and lead to metabolic generation of reactive oxygen species (Narayanan et al, 1997). Lehnert and Goodwin (1997) reported a short-lived factor generated from α irradiated culture medium containing serum and a longer-lived factor generated from irradiated fibroblasts. Azzam et al (1998) showed that the expression levels of TP53, CDKN1A, CDC2, CCNB1 and RAD51 are significantly modulated in human fibroblast cell lines that were irradiated with very low fluences of α particles. While the name is not perhaps ideal, all these effects are collectively known as 'radiation induced bystander effects'. Our group has shown bystander effects when the medium from epithelial cells irradiated with γ rays is transferred to cultures that have not been irradiated (Mothersill and Seymour, 1997, 1998). This irradiated cell conditioned medium (ICCM) can reduce clonogenic survival and increase the incidence of apoptosis in cells that never sustained any irradiation. The effect is dependent on the cell number present at the time of irradiation, strongly suggesting the production of a molecule by the irradiated cell. The factor is stable when frozen to –20°C but destroyed on heating to 70°C. Holding the cells on ice during and post irradiation prevents production of the bystander effect in cells receiving the ICCM (Mothersill and Seymour, 1998).

Irradiation of cells with a microbeam, which permits the traversal of one cell or part of a cell in a field with a proton beam, has also been used as a technique to study the bystander effect. The data show that effects of single cell irradiation are not limited to the exposed cell but affect other cells in the vicinity. Mutation, chromosome aberration and protein induction, have all been shown in cells distant from the target cell (Prise et al, 1998; Belyakov et al, 1999; Wu et al, 1999). The mechanism is unclear as is the nature of the substance or signal. The microbeam data are similar to the low LET data in that manifestation of the effect does not require cell to cell contact. There is conflicting evidence concerning the role of cell to cell contact. In the papers produced by Azzam et al (1999) and Nagasawa and Little (in press), there is clear evidence for a role of gap junctional intercellular communication (GJIC), while the gamma ray effect clearly does not require cell–cell contact, either during generation of the signal or in the recipient cultures. In fact, the cell killing effect is slightly enhanced if GJIC is inhibited prior to irradiation (Mothersill and Seymour, 1998). All this suggests that the mechanism involves active metabolic processes and that a cell-derived factor or signal is released into the medium from cells during irradiation.

This plus the fact that ICCM exposed cultures showed high numbers of apoptotic bodies (Mothersill and Seymour, 1998) led us to seek evidence for production of apoptotic signals by

*Received 7 March 2000*
*Revised 23 June 2000*
*Accepted 28 June 2000*

*Correspondence to:* FM Lyng irradiated cells that would appear in the medium and initiate apoptosis in unexposed cells. To investigate this it was decided to look at the ability of ICCM to induce early events in the apoptotic cascade. ICCM generated from human immortalized keratinocytes was added to keratinocytes that had never been irradiated. The effect was monitored using mobilization of intracellular calcium, loss of mitochondrial membrane potential and increase in reactive oxygen species as markers of apoptosis over a 24 hour period after exposure. All these events have been clearly linked with induction of apoptosis (Ojcius et al, 1991; Garland and Halestrap, 1997; Kroemer et al, 1997; Green and Reed, 1998). In addition, we have previously shown that treatment with anti-oxidants, L-Lactate and L-Deprenyl, prevented the bystander effect in ICCM exposed HPV-G cells (Mothersill et al, 2000). Treatment with cyclosporin A, which inhibits the collapse of mitochondrial membrane potential, and the ICE inhibitor (AC-YVAD-Cmk), which inhibits selected caspases involved in the apoptotic cascade, also reduced or prevented the bystander effect in these cells.

### METHODS

### Cell culture

A human keratinocyte cell line supplied as a gift by J Di Paolo, NIH, Bethesda, was used for most experiments. This line was originally immortalized by transfection with the HPV 16 virus (Pirisi et al, 1988). It is p53 null due to expression of E6 protein by the virus but grows in culture to form a characteristic monolayer of cobblestone-like keratinocytes. These display contact inhibition and gap junctional intercellular communication. This cell line was chosen because it expresses a reliable bystander effect in our hands that is constant over a range of radiation doses. No normal human epithelial cell line exists unless p53 is compromised in some way and p53 null was considered preferable to p53 mutant, where the nature of the mutation might be unknown or its interaction with the bystander effect might be suspect.

HPV keratinocytes were cultured in Dulbecco's MEM:F12 (1:1) containing 7% fetal calf serum, 5 ml penicillin streptomycin solution, 25 mM HEPES buffer and 1 μg/ml hydrocortisone (all from Gibco Biocult Ltd, Irvine; Scotland) and were maintained in an incubator at 37°C in an atmosphere of 5% $CO_2$ in air. Subculture was routinely performed using a 1:1 solution of 0.25% trypsin and 1 mM EDTA in Earle's balanced salt solution at 37°C.

### Chemicals

2,7-dichlorofluoresin diacetate, rhodamine 123 (Sigma), Fluo 3 and Fura Red acetoxymethyl (AM) ester (Molecular Probes) were dissolved in DMSO. All dilutions were made in buffer solutions so that the final concentration of DMSO was less than 0.1%. This volume of DMSO was added to controls and was shown to have no effect.

### Clonogenic assay

Subconfluent flasks that had received a medium change the previous day were chosen. Cells were removed from the flask using trypsin/EDTA solution. When the cells had detached they were resuspended in medium, pipetted gently to produce a single cell suspension and an aliquot was counted using a Coulter counter model $D_N$ set at a threshold calibrated for the cell line using a haemocytometer. Appropriate cell numbers were plated for survival using the clonogenic assay technique of Puck and Marcus (1956). Flasks destined to donate medium were plated with cell numbers in the region of $2 \times 10^5$. Medium was harvested 1 hour post irradiation which took place 6 hours after plating. The harvested medium was transferred to cultures containing cloning densities of cells (approx. 600 for HPV-G cells) set up at the same time as the donors. Controls for transfer of unirradiated medium from densely seeded cultures to cultures seeded at cloning densities were also always included as were simple plating efficiency controls. Cultures were incubated in 5 ml of culture medium in 25 $cm^2$, 40 ml flasks (Nunclon, Denmark), in a humidified 37°C incubator in an atmosphere of 5% $CO_2$ in air. After 9 days, cultures were stained with carbol fuchsin and macroscopic colonies, of greater than 50 cells, were counted. The percentage survival was corrected for the appropriate control plating efficiency.

### Irradiation

Irradiation took place 6 hours after plating the cells. Cultures were sealed and irradiated at room temperature using a cobalt 60 teletherapy unit delivering approximately 2.0 Gy/min during the time period of these experiments. The source to flask distance was 80 centimetres and the field size was 30 × 30 centimetres. Flasks were returned to the incubator immediately after irradiation. Control flasks were removed from the incubator and handled under the same conditions as the irradiated cells.

### Medium transfer and generation of donor medium for experiments

The technique used has been described in detail in Mothersill and Seymour (1997). Briefly, medium was poured off donor flasks one hour after irradiation. The medium was filtered through a 0.22 μm filter used to sterilize solutions, to ensure that no cells could still be present in the transferred medium. This was also confirmed by examination of aliquots of medium under the microscope. Culture medium was then removed from the flasks designated to receive irradiated medium and the filtrate was immediately added to these recipient flasks. A medium change of unirradiated but similarly filtered medium from unirradiated donor flasks seeded at the donor density of approx. 300 000 cells per flask was given to controls at the same time. Standard plating efficiency controls were also set up. There was never a significant difference between these two controls. Standard clonogenic survival points following irradiation were also always included, with and without a medium change at the appropriate time. No effect of changing the medium was found for any of the cell lines. The donor medium generated as described in this paragraph is referred to as ICCM (Irradiated Cell Conditioned Medium).

### Measurement of apoptosis

This was recorded in 5 random fields containing approximately 100 cells each, in each of three replicate flasks per treatment group. A cell was scored as apoptotic when it had a shrunken, dense morphology and a fragmented nucleus. The morphology is quite characteristic and was confirmed in some cases using electron microscopy. Previous data in the laboratory (unpublished)

 © 2000 Cancer Research Campaign confirmed that in this cell line the peak score of apoptotic cells occurred 48 hours after irradiation.

### Ratiometric measurement of calcium

Intracellular calcium levels were measured using two visible wavelength calcium sensitive dyes, Fluo 3 and Fura Red. Fluo 3 exhibits an increase in green fluorescence upon binding to calcium whereas Fura Red exhibits a decrease in red fluorescence upon binding to calcium. The ratio Fluo 3/Fura Red is a good indication of intracellular calcium levels (Lipp and Niggli 1993). Cultures were washed twice with a buffer containing 130 mM NaCl, 5 mM KCl, 1 mM $Na_2HPO_4$, 1 mM $CaCl_2$, 1 mM $MgCl_2$ and 25 mM HEPES (pH 7.4). Cells were loaded with the calcium sensitive dyes by incubation with 3 μM Fluo-3 and 3 μM Fura Red AM esters for 1 hour in the buffer at 37°C. Subsequently, the cultures were washed three times with buffer. Fluo 3 and Fura Red were excited at 488 nm and fluorescence emissions at 525 nm and 660 nm were recorded simultaneously using a Bio Rad 1024 confocal microscope. Ratio images and time course data of the Fluo 3/Fura Red fluorescence emissions were recorded every 2 seconds. ICCM was added after 60 seconds when a stable baseline had been established.

### Measurement of mitochondrial membrane potential

Mitochondrial membrane potential was measured using rhodamine 123. Rhodamine 123 is a green fluorescent dye that accumulates in active mitochondria with high membrane potentials (Ferlini et al, 1996). Cultures were washed twice with a buffer containing 130 mM NaCl, 5 mM KCl, 1 mM $Na_2HPO_4$, 1 mM $CaCl_2$, 1 mM $CaCl_2$, 1 mM $MgCl_2$ and 25 mM HEPES (pH 7.4). Cells were loaded with 5 μM rhodamine 123 for 30 min in the buffer at 37°C. Subsequently, the cultures were washed three times with buffer. Rhodamine 123 was excited at 488 nm and fluorescence emission at 525 nm was recorded using a Bio Rad 1024 confocal microscope. The percentage of fluorescently labelled cells in a defined area was used as a quantitative measure of mitochondrial membrane potential in control and treated cells.

### Measurement of reactive oxygen species

Induction of reactive oxygen species was measured using 2,7-dichlorofluorescein diacetate. 2,7-dichlorofluorescein diacetate emits green fluorescence when oxidized by reactive oxygen species (Yang, 1998). Cultures were washed twice with a buffer containing 130 mM NaCl, 5 mM KCl, 1 mM $Na_2HPO_4$, 1 mM $CaCl_2$, 1 mM $MgCl_2$ and 25 mM HEPES (pH 7.4). Cells were loaded with 5 μM 2,7-dichlorofluorescein diacetate for 30 min in the buffer at 37°C. Subsequently, the cultures were washed three times with buffer. 2,7-dichlorofluorescein diacetate was excited at 488 nm and fluorescence emission at 525 nm was recorded using a Bio Rad 1024 confocal microscope. The percentage of fluorescently labelled cells in a defined area was used as a quantitative measure of oxidative damage in control and treated cells.

### Statistical analysis

Measurements of $[Ca^{2+}]_i$, clonogenic survival and apoptotic cells are presented as mean values ± S.E. of 3 independent experiments each performed in triplicate. Significance of differences was determined by a student's unpaired *t*-test and the differences were considered significant if $P \leq 0.05$.

Fluorescence images of mitochondrial membrane potential and reactive oxygen species were recorded from samples from 3 independent experiments each performed in triplicate.

**Table 1** Clonogenic surviving fractions for human keratinocyte cells exposed to the radiation dose or to ICCM

| Dose | Radiation | ICCM |
|---|---|---|
| 0 Gy | 100 ± 3.38 | 100 ± 5.55 |
| 0.5 Gy | 59.8 ± 3.93 | 59.9 ± 2.65 |
| 5 Gy | 20.4 ± 0.46 | 66.3 ± 1.9 |

Errors are the standard error of the mean for $n$ = 3. Actual plating efficiencies for controls were: Radiation group: 32.8 ± 1:1, ICCM recipient group; 32.04 ± 1.78

**Table 2** Percentage of HPV-G cells with apoptotic morphology detected in cultures 48 h following direct irradiation or exposure to medium from irradiated cells (ICCM)

| Dose | Radiation | ICCM |
|---|---|---|
| 0 Gy | 1.3 ± 0.08 | 0.9 ± 0.1 |
| 0.5 Gy | 14.7 ± 2.1 | 13.9 ± 2.6 |
| 5.0 Gy | 12.6 ± 1.8 | 15.0 ± 2.5 |

### RESULTS

### Clonogenic survival following direct irradiation or treatment with ICCM

Table 1 shows the actual clonogenic survival data for cells in these experiments treated with radiation or ICCM. The direct radiation dose and ICCM both had the same effect on survival at 0.5 Gy. At 5 Gy the direct irradiation was more toxic than the ICCM which showed no statistically significant change in toxicity as a result of being generated by cells irradiated to 0.5 or 5 Gy. It is also clear from the table that the controls for ICCM exposed cultures which received the filtered medium from densely seeded cultures have the same plating efficiency as the controls for the irradiated group that were seeded at cloning density (approx 500 cells).

### Apoptosis

The percentage of cells showing apoptotic morphology 48 h following direct irradiation or exposure to medium from irradiated cells (ICCM) is shown in Table 2. The direct irradiation and ICCM both resulted in increased apoptosis at 0.5 Gy and 5 Gy.

### Intracellular calcium

Rapid calcium fluxes (within 30 s) were observed following addition of ICCM. Figure 1 shows a rapid and transient increase in calcium levels following addition of 0.5 Gy ICCM. Figure 2 shows a similar response following addition of 5 Gy ICCM. There was no significant difference between the response to 0.5 Gy ICCM and to 5 Gy ICCM. Ratio images of calcium levels before

© 2000 Cancer Research Campaign

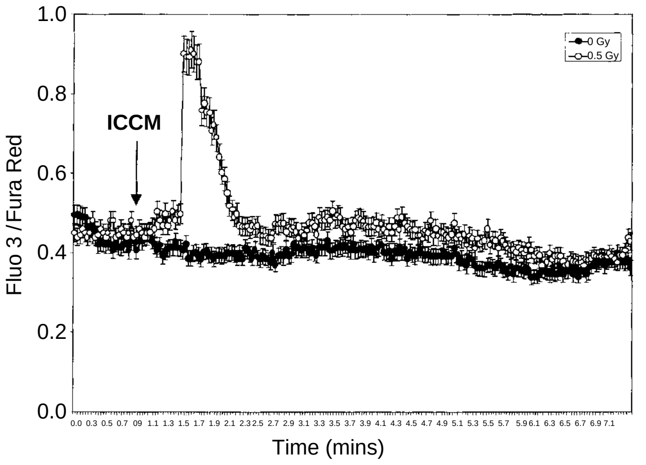

**Figure 1** Intracellular calcium levels in HPV-G cells after addition of medium from unirradiated cells (0 Gy ICCM) and medium from irradiated cells (0.5 Gy ICCM). ICCM was added at the time indicated by the arrow. The ratio of fluorescence emissions from the calcium sensitive dyes Fluo 3 and Fura Red provides an indication of intracellular calcium levels

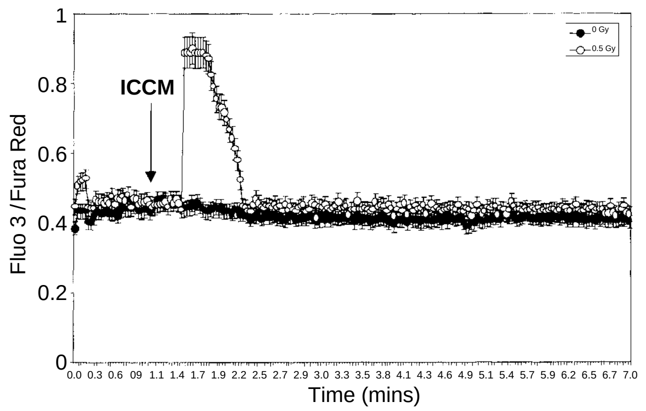

**Figure 2** Intracellular calcium levels in HPV-G cells after addition of medium from unirradiated cells (0 Gy ICCM) and medium from irradiated cells (5 Gy ICCM)

and after addition of 0.5 Gy ICCM and 5 Gy ICCM are shown in Figure 3. The images are colour coded for calcium levels; blue indicates low levels of calcium while green, yellow and red indicate progressively higher levels of calcium. There was no change in intracellular calcium levels following addition of control medium or of medium from densely seeded but unirradiated cells (0 Gy ICCM) (Figure 3).

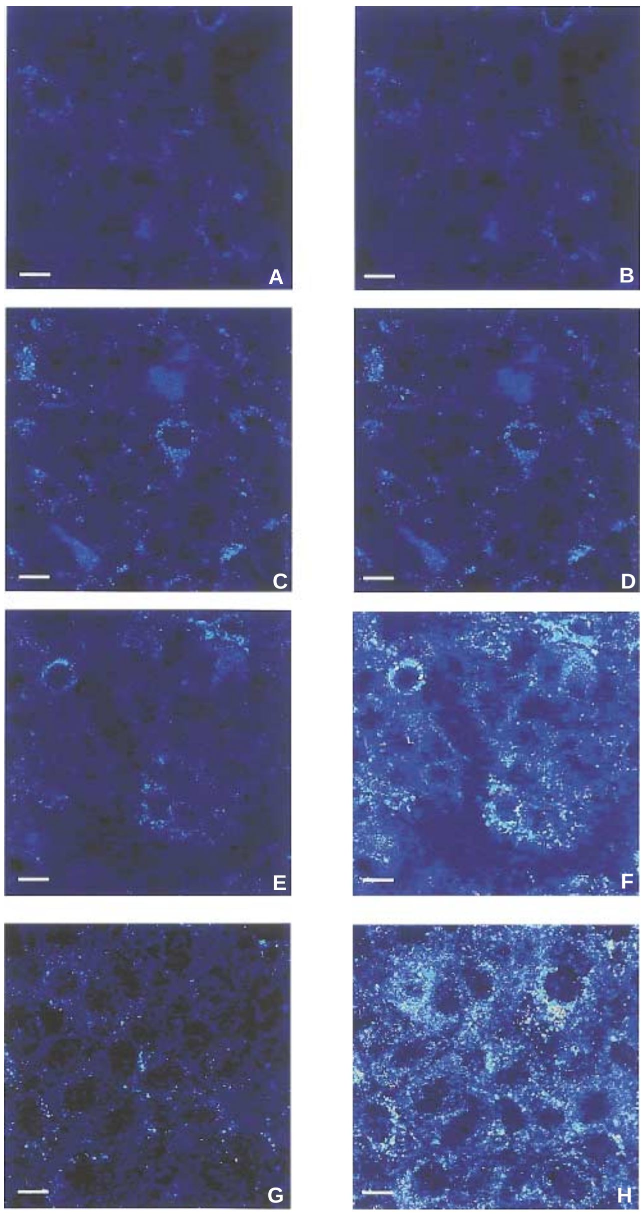

**Figure 3** Ratio images of calcium levels in HPV-G cells (**A**) before and (**B**) 30 s after addition of control medium (fresh medium), (**C**) before and (**D**) 30 s after addition of medium from unirradiated cells (0 Gy ICCM), (**E**) before and (**F**) 30 s after addition of medium from irradiated cells (0.5 Gy ICCM) and (**G**) before and (**H**) 30 s after addition of medium from irradiated cells (5 Gy ICCM). Blue indicates low levels of calcium while green, yellow and red indicate progressively higher levels of calcium. Bar = 5 μm

### Mitochondrial membrane potential

Mitochondria with high membrane potentials were observed in control cells and in cells treated with 0 Gy ICCM (Figure 4B). No change in mitochondrial membrane potential was observed at 30 s or 1 h following addition of medium from irradiated cells (Figure 4D) but a decrease in fluorescence and more unspecific staining was observed at 6, 12 and 24 hours after addition (Figure 4E, F). This suggests a decrease in mitochondrial membrane potential. There was no significant difference between the response to 0.5 Gy ICCM and to 5 Gy ICCM. Table 3 shows the percentage of fluorescent cells in a defined area as a quantitative indicator of membrane potential in cells exposed to ICCM.

### Reactive oxygen species

No fluorescence was observed in control cells or cells treated with medium from unirradiated cells (0 Gy ICCM) (Figure 5B). No fluorescence was observed at 30 s or 1 h following addition of medium from irradiated cells (Figure 5D) but an increase in fluorescence was observed at 6, 12 and 24 hours after addition (Figure 5E, F). This suggests an increase in reactive oxygen species. There was no significant difference between the response to 0.5 Gy ICCM and to 5 Gy ICCM. Table 4 shows the percentage of

© 2000 Cancer Research Campaign

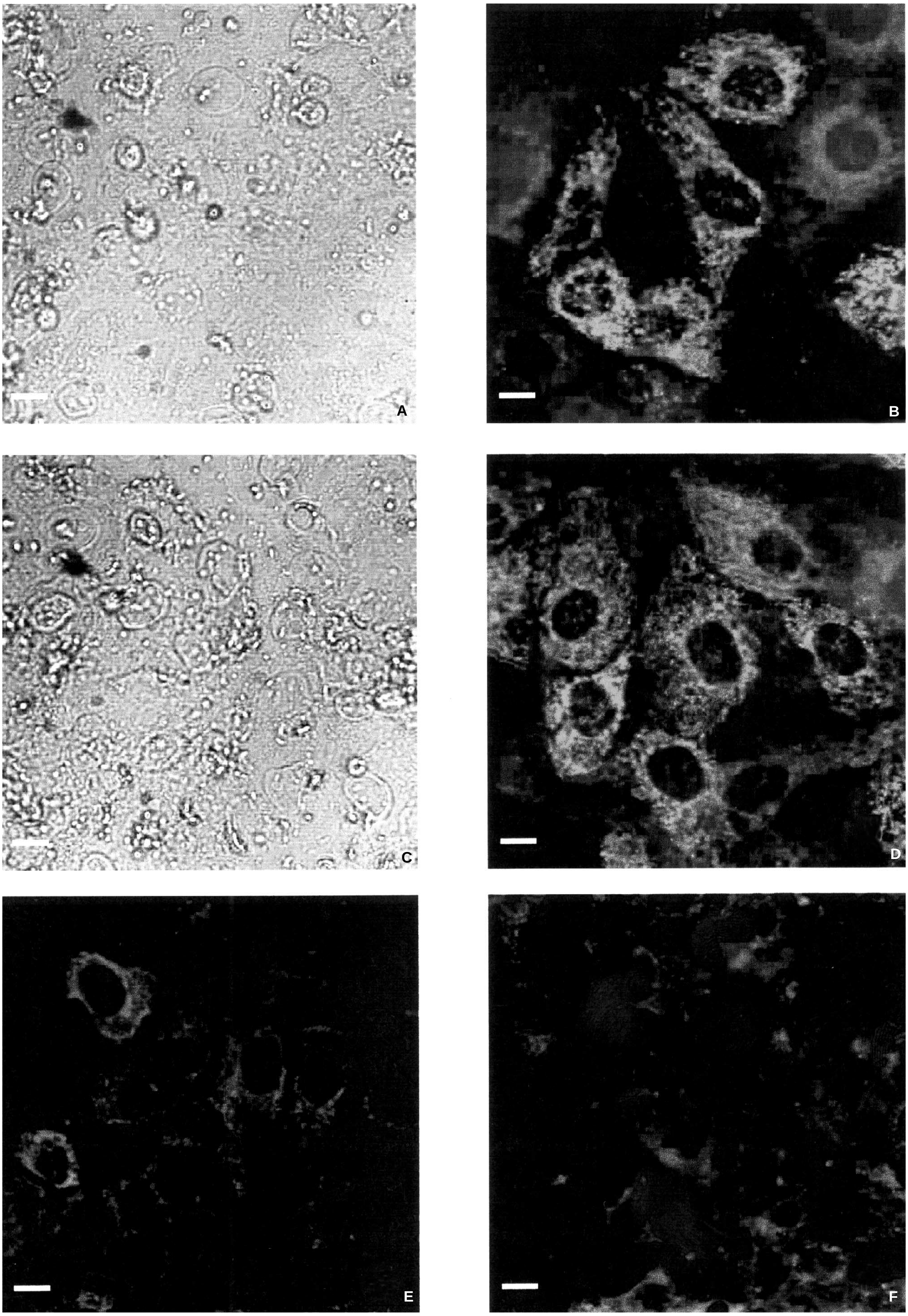

**Figure 4** Transmitted light images of (**A**) control HPV-G cells and (**C**) HPV-G cells treated with ICCM. Fluorescence images showing the level of mitochondrial membrane potential in (**B**) control HPV-G cells and HPV-G cells (**D**) 1 hour after addition, (**E**) 6 hours after addition and (**F**) 12 hours after addition of 0.5 Gy ICCM. A decrease in green fluorescence is indicative of a loss of membrane potential. Bar = 5 μm

*© 2000 Cancer Research Campaign*

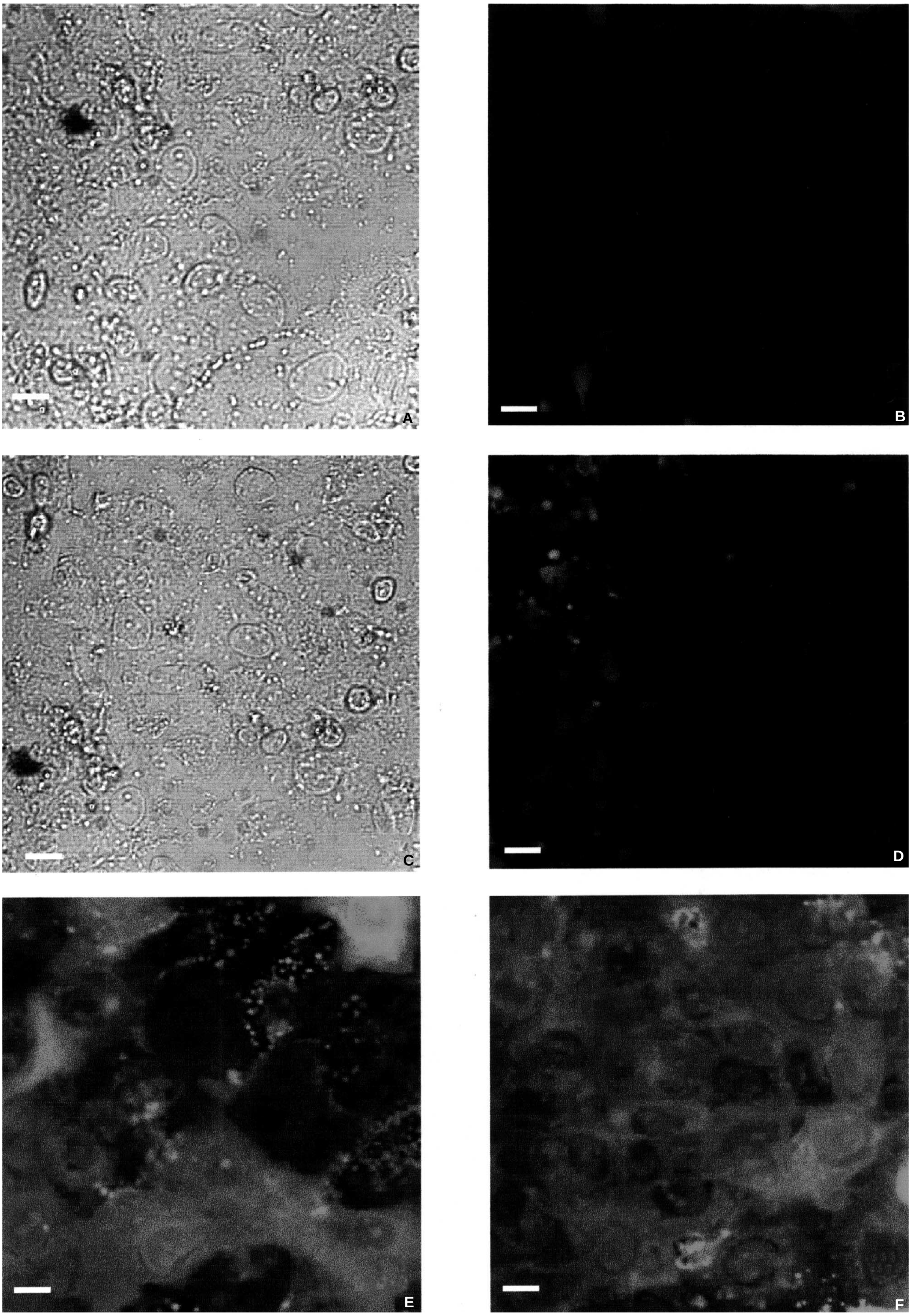

**Figure 5** Transmitted light images of (**A**) control HPV-G cells and (**C**) HPV-G cells treated with ICCM. Fluorescence images showing the level of reactive oxygen species in (**B**) control HPV-G cells and HPV-G cells (**D**) 1 hour after addition, (**E**) 6 hours after addition and (**F**) 12 hours after addition of 0.5 Gy ICCM. An increase in green fluorescence is indicative of an increase in reactive oxygen species. Bar = 5 µm

 *© 2000 Cancer Research Campaign*

**Table 3** Percentage of cells showing rhodamine 123 fluorescence (indicator of mitochondrial membrane potential) following exposure to medium from irradiated cells (ICCM) at various time points

| Time | 0 Gy | 0.5 Gy | 5 Gy |
|---|---|---|---|
| 30 sec | 62.56 ± 3.58 | 60.44 ± 4.32 | 64.11 ± 4.88 |
| 1 hour | 61. 23 ± 3.76 | 66.9 ± 2.85 | 63.29 ± 3.55 |
| 6 hours | 60.33 ± 2.99 | 20.65 ± 0.89 | 20.78 ± 0.96 |
| 12 hours | 62.96 ± 3.80 | 22.77 ± 1.44 | 21.86 ± 1.39 |
| 24 hours | 60.45 ± 2.55 | 20.79 ± 1.22 | 20.93 ± 0.90 |

**Table 4** Percentage of cells showing dichlorofluorescin diacetate fluorescence (indicator of oxidative damage) following exposure to medium from irradiated cells (ICCM) at various time points

| Time | 0 Gy | 0.5 Gy | 5 Gy |
|---|---|---|---|
| 30 sec | 7.32 ± 0.34 | 6.78 ± 0.49 | 6.91 ± 0.58 |
| 1 hour | 6.99 ± 0.45 | 67.84 ± 2.84 | 69.22 ± 2.76 |
| 6 hours | 6.59 ± 0.36 | 75.45 ± 3.12 | 74.88 ± 2.88 |
| 12 hours | 7.08 ± 0.41 | 73.11 ± 2.43 | 71.96 ± 2.49 |
| 24 hours | 6.95 ± 0.25 | 74.82 ± 2.75 | 73.98 ± 1.80 |

fluorescent cells in a defined area as a quantitative indicator of oxidative damage.

### DISCUSSION

The fact that cells exposed to high or low LET radiations produce a factor or signal that can influence their neighbours is not now in doubt. The mechanisms by which the signal is produced and transduced are however, largely unknown. There is some evidence that apoptotic death is a prominent feature of cultures demonstrating bystander effects and this is supported by the data presented in this paper. Kroemer et al (1997) showed that one of the first observable effects in the pathway leading to apoptosis was a change in the membrane permeability in the mitochondria, which resulted in the release of apoptosis inducing factor from the mitochondria. Mitochondrial depolarization is thought to be associated with the early stage of apoptosis. Changes in the membrane potential are presumed to be due to the opening of the mitochondrial permeability transition pores that play a central role in apoptosis (Green and Reed, 1998). Intracellular calcium fluxes are also thought to be involved in the induction of apoptosis (Ojcius et al, 1991). An increase in reactive oxygen species has also been linked to the initiation of the apoptotic cascade (Garland and Halestrap, 1997). Intracellular calcium fluxes, loss of mitochondrial membrane potential and increases in reactive oxygen species have all been shown in this paper to be induced in p53 null immortal human epithelial cells exposed to ICCM. A reduction in clonogenic survival and evidence of apoptosis have also been observed. While it would have been interesting to investigate whether direct irradiation induces calcium fluxes, loss of mitochondrial membrane potential and increases in reactive oxygen species, it was not possible within the time frame of the experiment (30 seconds for calcium fluxes).

However, we have previously shown later stages of the apoptosis pathway in irradiated and ICCM exposed HPV-G cells (Mothersill and Seymour, 1998), and radiation is a well known inducer of apoptosis in many cell lines. Our aim was to characterize the signal produced by irradiated cells rather than to investigate the effect of direct radiation exposure. Seymour and Mothersill (2000) have investigated cell killing by direct radiation and the bystander effect over a dose range of 0.01–5 Gy. Their data show that doses of 0.01–0.5 Gy only induce clonogenic death by the bystander effect, the magnitude of the effect is relatively constant and appears to saturate at doses in the range of 0.03–0.05 Gy. After doses greater than 0.5 Gy, the clonogenic death curves are the result of a dose dependent non-bystander effect and a dose independent bystander effect.

The calcium flux observed is remarkably fast (within 30 secs) and transient (lasting only 30–40 s) and must result from reception of a signal contained in the ICCM. It is interesting that the changes in mitochondrial membrane permeability persist for at least 24 hours. Changes in the expression of oxidative stress also persisted. Both oxidative stress and miochondrial abnormalities have been suggested as important factors in the production of persistent, non-clonal genomic instability in cells (Clutton et al, 1996). This group showed that irradiated cells that survive have a persistently elevated production of toxic oxygen radicals. They proposed that the de novo production of new aberrations in the descendants of irradiated cells was due to oxidative damage but had no explanation for why the oxygen radical generation was persistently elevated. Recent data from several laboratories (Seymour and Mothersill, 1997; Lorimore et al, 1998; Azzam et al, 1999; Wu et al, 1999) have shown that all the endpoints which characterize ‘genomic instability’, can in fact be induced in cells that were never directly exposed to irradiation. Wu et al (1999) and Azzam et al (1999) showed mutations in cells that were not traversed by a radiation track. Lorimore et al (1998) showed that persistent chromosomal aberrations following alpha particle irradiation were found in the parts of the culture which were not ever hit by a particle. Seymour and Mothersill (1997) showed that cultures receiving ICCM showed high levels of lethal mutations (delayed death) in the distant progeny. They also showed that the same amount of delayed death could be induced by the dose of radiation to the progenitors or by the exposure of the progenitors to ICCM (Seymour and Mothersill, 1999). On the basis of all these data, it now appears that the phenomenon of radiation induced genomic instability may in fact be induced by the bystander factor. A possible mechanism could involve an induction of a persistent oxidative stress response, as proposed by Clutton et al (1996), which can result in the production of reactive oxygen species (ROS) leading to oxidative damage (chromosome breaks/mutations). Persistent initiation of the apoptotic cascade through a relatively long-term change in mitochondrial membrane permeability would be consistent with findings by Lyng et al (1996) which showed persistent apoptosis in distant progeny of irradiated cells.

Previous work by our group and Wright’s group identified a genetic component in the induction of instability phenomena in irradiated cells. The data in Kadhim et al (1997) and Mothersill et al (1999) suggest that genetic factors leading to expression of chromosomal instability may be counteracted by other factors, which cause a long-term programmed cell death response to radiation. The data in this paper, showing that ICCM is able, in the long-term, to induce ROS, which was shown by Clutton et al (1996) to lead to chromosomal instability, and also to initiate the apoptotic cascade, means that the signal produces a response in cells which can lead to death or to persistence of damage. The fate of the cell and ultimately, the tissue or organism, may well be determined by the genetic make-up of the individual and

© 2000 Cancer Research Campaign pre-existing epigenetic factors which determine whether the cell undergoes programmed cell death or lives to produce progeny.

### ACKNOWLEDGEMENTS

We are grateful to Professor Brian Harvey, University College Cork, for access to the Bio Rad 1024 confocal microscope and to St Luke's Hospital, Dublin for continued access to the cobalt-60 source. We wish to thank the Irish Cancer Research Advancement Board who supported this work.

### REFERENCES

Azzam EI, de Toledo SM, Gooding T and Little JB (1998) Intercellular communication is involved in the bystander regulation of gene expression in human cells exposed to very low fluences of alpha particles. *Radiation Research* **150**: 497–504

Belyakov OV, Prise KM, Mothersill C, Folkard M and Michael BD (1999) Studies of bystander effects in primary urothelial cells using a charged particle microbeam. *Radiation Research* (in press)

Clutton SM, Townsend KMS, Walker C, Ansell JD and Wright EG (1996) Radiation induced genomic instability and persisting oxidative stress in primary bone marrow cultures. *Carcinogenesis* **17**: 1633–1639

Deshpande A, Goodwin EH, Bailey SM, Marrone BL and Lehnert BE (1996) Alpha-particle-induced sister chromatid exchange in normal human lung fibroblasts: evidence for an extra-nuclear target. *Radiation Research* **145**: 260–267

Emerit I, Filipe P, Meunier P, Auclair C, Freitas J, Deroussent A, Gouyette A and Fernandes A (1967) Clastogenic activity in the plasma of scleroderma patients: a biomarker of oxidative stress. *Dermatology* **194**: 140–146

Ferlini C, Di Cesare S, Rainaldi G, Malorni W, Samoggia P, Biselli R and Fattorossi A (1996) Flow cytometric analysis of the early phases of apoptosis by cellular and nuclear techniques. *Cytometry* **24**: 106–115

Garland JM and Halestrap A (1997) Energy metabolism during apoptosis. *J Biol Chem* **272**: 4680

Green DR and Reed JC (1998) Mitochondria and apoptosis. *Science* **281**: 1309–1312

Kroemer G, Zamzami N and Susin SA (1997) Mitochondrial control of apoptosis. *Immunology Today* **18**: 44–51

Lehnert BE and Goodwin EH (1997) Extracellular factor(s) following exposure to alpha-particles can cause sister chromatid exchanges in normal human cells. *Cancer Research* **57**: 2164–2171

Lloyd DC and Moquet JE (1985) The clastogenic effect of irradiated human plasma. *Int J Radiat Biol* **47**: 433–444

Lyng FM, O'Reilly S, Cottell DC, Seymour CB and Mothersill C (1996) Persistent expression of morphological abnormalities in the distant progeny of irradiated cells. *Radiation and Environmental Biophysics* **35**: 273–283

Lorimore SA, Kadhim MA, Pocock DA, Papworth D, Stevens DL, Goodhead, DT and Wright EG (1998) Chromosomal instability in the descendants of unirradiated surviving cells after α-particle irradiation. *Proceedings of the National Academy of Sciences USA* **95**: 5730–5733

Mothersill C and Seymour CB (1997) Medium from irradiated human epithelial cells but not human fibroblasts reduces the clonogenic survival of unirradiated cells. *International Journal of Radiation Biology* **71**: 421–427

Mothersill C and Seymour CB (1998) Cell–cell contact during gamma irradiation is not required to induce a bystander effect in normal human keratinocytes: evidence for release during irradiation of a signal controlling survival into the medium. *Radiation Research* **149**: 256–262

Mothersill C, Harney J, Lyng F, Cottell D, Parsons K, Murphy D and Seymour CB (1995) Primary explants of human uroepithelium show an unusual response to low-dose irradiation with cobalt-60 gamma rays. *Radiat Res* **142**: 181–187

Mothersill C, O'Malley K, Murphy D, Seymour C, Lorimore S and Wright EG (1999) Identification and characterisation of three subtypes of radiation response in normal human urothelial cultures exposed to ionizing radiation. *Carcinogenesis* **20**: 12

Mothersill C, Stamato TD, Perez ML, Cummins R, Mooney R and Seymour CB (2000) Involvement of energy metabolism in the production of "bystander effects" by radiation, *Br J Cancer* **82**(10): 1740–1746

Nagasawa H and Little JB (1992) Induction of sister chromatid exchanges by extremely low doses of alpha particles. *Cancer Research* **52**: 6394–6396

Nagasawa H and Little JB (1999) Unexpected sensitivity to the induction of mutations by very low doses of alphaparticle radiation: evidence for a bystander effect. *Radiation Research* (in press)

Narayanan PK, Goodwin EH and Lehnert BE (1997) α particles initiate biological production of superoxide anions and hydrogen peroxide in human cells. *Cancer Research* **57**: 3963–3971

Ojcius DM, Zychlinsky A, Zheng LM and Young JD (1991) Ionophore induced apoptosis: role of DNA fragmentation and calcium fluxes. *Exp Cell Res* **197**: 43

Pirisi L, Yasumoto S, Feller S, Doniger J and DiPaolo J (1988) Transformation of human fibroblasts and keratinocytes with human papillomavirus type 16 DNA. *Journal of Virology* **61**: 1061–1066

Prise KM, Belyakov OV, Folkard M and Michael BD (1998) Studies of bystander effects in human fibroblasts using a charged particle microbeam. *Int J Radiat Biol* **74**: 793–798

Puck TT and Marcus PI (1956) Action of X-rays on mammalian cells. *Journal of Experimental Medicine* **103**: 653–666

Seymour CB and Mothersill C (1997) Delayed expression of lethal mutations and genomic instability in the progeny of human epithelial cells that survived in a bystander killing environment. *Radiat Oncol Investig* **5**: 106–110

Seymour CB and Mothersill C (2000) Relative contribution of bystander and targeted cell killing to the low dose region of the radiation dose response curve. *Radiation Research* **153**(5): 508–511

Wu LJ, Randers-Pehrson G, Xu A, Waldren CA, Geard CR, Yu Z and Hei TK (1999) Targeted cytoplasmic irradiation with alpha particles induces mutations in mammalian cells. *Proc Natl Acad Sci USA* **96**: 4959–4964

Yang HW (1998) Detection of reactive oxygen species (ROS) and apoptosis in human fragmented embryos. *Human Reprod* **13**: 998

 *© 2000 Cancer Research Campaign*
